# BACE1 Function and Inhibition: Implications of Intervention in the Amyloid Pathway of Alzheimer’s Disease Pathology

**DOI:** 10.3390/molecules22101723

**Published:** 2017-10-13

**Authors:** Gerald Koelsch

**Affiliations:** CoMentis, Inc., South San Francisco, CA 94080, USA; geraldkoelschcomentis@gmail.com

**Keywords:** Alzheimer’s disease, amyloid hypothesis, BACE1, beta secretase, pharmacology

## Abstract

Alzheimer’s disease (AD) is a fatal progressive neurodegenerative disorder characterized by increasing loss in memory, cognition, and function of daily living. Among the many pathologic events observed in the progression of AD, changes in amyloid β peptide (Aβ) metabolism proceed fastest, and precede clinical symptoms. BACE1 (β-secretase 1) catalyzes the initial cleavage of the amyloid precursor protein to generate Aβ. Therefore inhibition of BACE1 activity could block one of the earliest pathologic events in AD. However, therapeutic BACE1 inhibition to block Aβ production may need to be balanced with possible effects that might result from diminished physiologic functions BACE1, in particular processing of substrates involved in neuronal function of the brain and periphery. Potentials for beneficial or consequential effects resulting from pharmacologic inhibition of BACE1 are reviewed in context of ongoing clinical trials testing the effect of BACE1 candidate inhibitor drugs in AD populations.

## 1. Introduction

Alzheimer’s disease (AD) is a fatal progressive neurodegenerative disorder, slowly eroding memory, cognition, and functions of daily living, inevitably culminating in death from pneumonia and infectious diseases resulting from failure to thrive, loss of fine motor skills, and incapacitation. Treatment is limited to therapeutics that alleviate symptoms of memory loss, but are effective for a relatively short duration during and after which disease progression continues. Therefore much effort has been directed toward development of disease modifying therapeutics to slow or delay the progressive trajectory of AD.

Pathologically, AD is characterized by loss of neuronal brain mass and presence of insoluble particles visible microscopically as intracellular microfibrillary tangles and extracellular “starch-like” amyloid plaques. The principal composition of the latter of these two proteinopathies is the amyloid β (Aβ) peptide [1,2]. Landmark brain imaging and biomarker initiatives [3] examined these and other AD pathologies longitudinally, and models for pathologic events in AD have proceeded from the results (for example, [4]). Accumulation of Aβ is one of the earliest events in the disease process, occurring to near completion 10–20 years prior to the onset of memory loss and other clinical symptoms [5]. Evidence equivocally shows the bulk of amyloid pathology proceeds faster than all other events [4,6]. Therefore numerous therapies have been aimed at reducing amyloid, through agents acting directly on forms of Aβ or on its generation or metabolism.

The Aβ peptide present in amyloid plaque is approximately 36–43 amino acids in length, generated from the amyloid precursor protein (APP) by a series of proteolytic cleavages followed by a broad diversity of post-translational modifications (in this review are all collectively referred to as Aβ). Myriad Aβ peptides have a varied propensity to oligomerize and aggregate with other Aβ peptides and assemblies thereof, with diverse effects on cognitoxicity and neurotoxicity [1,7,8]. Aβ may alternatively participate in normal synaptic function [9,10,11,12]. Although generation and clearance of Aβ may be part of normal physiology, its overproduction or under-clearance may result in a shift in the “Aβ economy” [13] towards accumulation, and a slow attrition towards disease.

**Figure 1:**
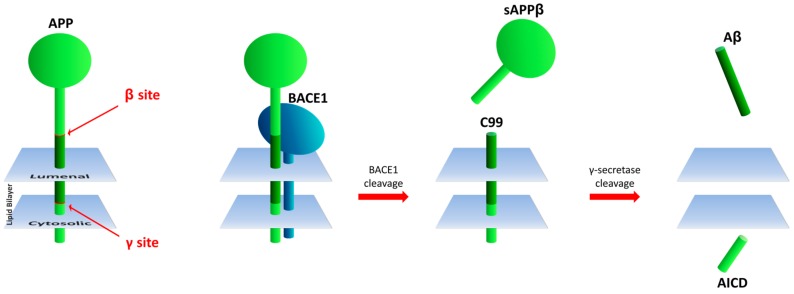
Processing of APP by BACE1 to generate Aβ. APP (left panel, green object) is depicted with the Aβ in darker green, with the β site and γ site in red. Cleavage by BACE1 (indigo object) generates soluble APPβ (sAPPβ) and C99, followed with cleavage by γ-secretase to generate Aβ and APP intracellular domain (AICD).

β-Secretase 1 (BACE1) catalyzes the rate-limiting, initial cleavage at the β site of APP [14] and is followed by sequential intra-membrane processing at multiple sites by γ-secretase in the generation of Aβ (Figure 1). Genetic changes resulting in amino acid differences at the β-cleavage site of APP result in increased or decreased BACE1 activity toward the APP substrate and are associated with early onset familial AD (FAD) or protection from disease, respectively [14,15]. Thus, BACE1 has been considered a relevant target for development of drugs to inhibit its secretase activity towards APP to thwart Aβ production and AD progression.

BACE1 has been implicated as the APP β-secretase [14,16]. Several BACE1 inhibitor drug candidates have advanced to Phase 3 clinical trials. A number of excellent reviews, many of which are cited herein, provide a comprehensive overview of BACE1 structure, function, biology and inhibition, highlighting the potential for both beneficial and deleterious effects resulting from depletion of BACE1. The aim of this review is to provide additional perspective and to revisit unique features of BACE1 structure and function.

## 2. BACE1 Characteristics

The β-site amyloid precursor protein cleaving enzyme 1 (BACE1), has been implicated as the APP β-site protease through its expression, intracellular colocalization with APP, and activities consistent with FAD mutations at the β site [14]. In this review, the β-secretase activity of only BACE1 is discussed, based on its suitability as a target for AD therapeutics, evidenced by the attainment of central Aβ reduction of greater than 90% in numerous trials (reviewed in Section 3.4 below).

BACE1 is an aspartic protease (aspartyl protease, aspartic acid protease, aspartic proteinase), one of several of members of the pepsin family that are endogenous to humans [17]. The prototypic pepsin was characterized having two aspartic acid residues in the active site, and other features common to all other members of the family [18]. The two active site aspartic acid residues are conserved within two Asp-(Thr/Ser)-Gly motifs in the amino acid sequence and in the three-dimensional structure are situated with two-fold rotational symmetry of one another, as are each of the globular sub-domains. BACE1, and its close homolog BACE2, possess additional lumenal, transmembrane and cytosolic domains extending beyond the predicted globular catalytic domain (memapsins, membrane anchored aspartic proteases of the pepsin family). Highly conserved among species, BACE1 with its unique structural attributes might be anticipated to perform specialized functions distinct from other general catalytic aspartic proteases.

### 2.1. Structural Attributes of BACE1

BACE1 is a type I transmembrane protein, a feature unique among other members of the pepsin family. A sequence of an additional 82 amino acids extends *C*-terminally to the homologous pepsin carboxyl terminus, and includes a lumenal extension, a hydrophobic region containing the transmembrane domain, and a cytosolic domain. Features of the globular homologous pepsin domain have been explored for structure based drug design [19,20,21,22,23,24,25,26,27,28,29]. Extended loops in this domain [30] were hypothesized as unique features with specialized function. Specificity for substrates at positions distal of the active site has been observed for BACE1 [31,32]. In particular allosteric modulation has been attributed to these sites [33], a feature unique to BACE1 among other aspartic proteases.

**Figure 2:**
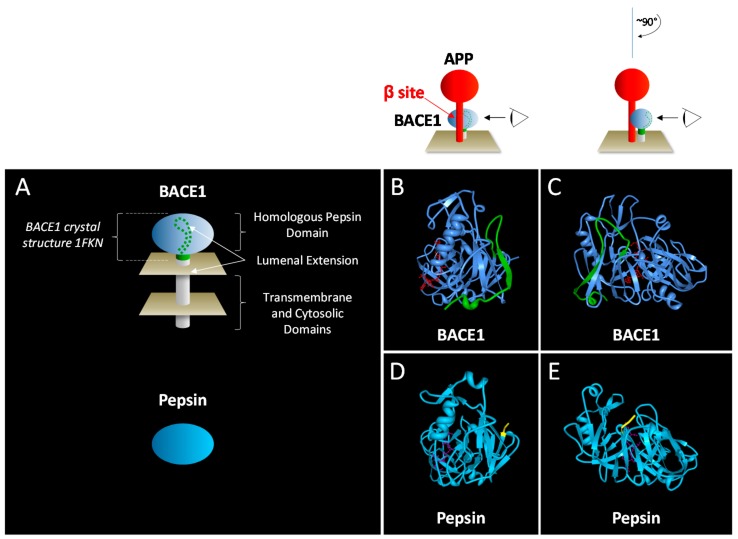
Lumenal extension of BACE1 interacts with the catalytic domain opposite of the active site. (**A**) Depiction scheme of BACE1 (**B**,**C**), Protein Data Bank code 1FKN [30]; and pepsin (**D**,**E**), Protein Data Bank code 1PSO [46]. The catalytic domain of BACE1 is depicted in light blue and a portion of BACE1 lumenal extension visible in the 1FKN ctrystal structure is depicted in green. (**B**,**D**) View of BACE1 (**B**) and pepsin (**D**) from side (BACE1 view orientation scheme above panel (**B**)). Ribbon diagram of BACE1 (color scheme as in panel (**A**)), with BACE1 inhibitor (atomic structure in red) marking location of the substrate APP; (**D**) Ribbon diagram of pepsin is viewed in approximately the same orientation as BACE1 in panel (**B**), with the carboxy terminal amino acids of pepsin (yellow) marking the homologous location of the *C*-terminal extension of BACE1. Pepsin inhibitor pepstatin A (violet atomic structure) is located in the pepsin active site. (**C**,**E**) View of BACE1 (**C**) and pepsin (**E**) from behind the active site (view orientation scheme above panel (**C**)). Color scheme as in panels (**B**,**D**). Ribbon diagrams created with the RSCB PDB Protein Workshop 4.2.0 Molecular Biology Toolkit [47].

The lumenal extension spans approximately 35 amino acids *C*-terminal to the homologous pepsin domain and contains secondary structures that interact directly with it (Table 1 footnote) by annealing to the conserved pepsin globular catalytic domain (Figure 2). Interestingly, both ends of the lumenal extension are attached by two disulfide bonds connecting directly to the catalytic domain [30]. These two disulfide bonds utilize one conserved and one non-conserved cysteine residue present in the pepsin domain (Table 1).

A short span of approximately 11 amino acids tether the BACE1 catalytic domain to the lipid bilayer membrane, *C*-terminally of the last disulfide bonded cysteine of the lumenal extension. Increases in Aβ production and BACE1 expression transcriptionally have been observed from cells exposed to hypoxic conditions [48,49,50]. Interestingly, disulfide bond formation may be impaired in hypoxic conditions [51]. Thus, it might be considered that the two disulfide bonds that attach both ends of the 35 amino acid lumenal domain might be incompletely formed under hypoxic conditions. Additionally, the secondary structures of the lumenal extension that interact with the conserved pepsin domain might be prone to reversibility, a prospect not unreasonable as the interactions are limited in number. Therefore, under hypoxic conditions, it might be hypothesized that an additional release of the BACE1 catalytic domain away from the lipid bilayer membrane would be possible by extending the tethering distance from 11 amino acids to 35 amino acids, presumably allowing a greater degree of freedom, increased access to substrate, and possibly increased activity.

Distal to a hydrophobic region spanning 26 amino acids that include the transmembrane domain, a further 21 amino acid sequence extends into the cytoplasmic (residues 494–501). This short span includes a dileucine sequence motif that interacts with the cytosolic domain of APP [52,53], and with Golgi-localized, gamma-ear containing, ADP-ribosylation factor binding proteins (GGA) 1 and 3 facilitate transport and sorting of BACE1 from endosomes, where it is associated with APP, to lysosomes to modulate amyloid processing [54,55,56].

A propeptide sequence between the signal peptide and the homologous amino terminus of pepsin is removed by a proprotein convertase after transit through the ER [57,58]. The BACE1 propeptide region lacks homology and features in common to other aspartic proteases, although propeptides of the pepsin family generally lack sequence conservation [59]. Propeptides of aspartic proteases may assist in folding and maintaining inactive states, and in some cases serve functional roles in sorting [60]. Propeptide processing (Table 1) is not required for BACE1 activity, but does result in an increase of activity [36]. Interestingly, BACE1 activity may be mediated also by the position of the “beta flap”, a β-sheet hairpin loop that looms above the active site [61].

A battery of post-translational modifications observed for BACE1 [45] are summarized in Table 1 and include several which contribute to its intracellular localization and specialized function [62] as well as its uniqueness as a member of the aspartic protease family.

### 2.2. BACE1 Activity and Native Substrates

BACE1 is present in endosomes and to a lesser extent in trans-Golgi network, compatible with its optimal activity in acidic environments [63], and localizes to lipid rafts, possibly implicated in amyloidogenic pathway of APP processing [64]. Events in the compartmental transit of BACE1 and its association with APP are complex [14]. BACE1 reaches the plasma membrane after synthesis, and is internalized by cholesterol and lipid dependent pathways [65,66]. Recycling of BACE1 within endosomal compartments has been implicated in maintaining amyloidogenic activity [67,68], in opposition to lysosomal destination of BACE1 where it is degraded [42,69]. Heritable molecular defects in secretory and endocytic pathways that regulate BACE1 processing of APP may increase Aβ production, further implicating BACE1 and amyloid in AD, and may be targeted for developing AD therapies [70]. Importantly, neuronal retrograde transport and somatic localization of BACE1 has been shown to be essential for limiting BACE1 activity and generation of Aβ in the synapse [71,72]. BACE1 inhibition would be expected to impact the end result of Aβ irrespective of cellular trafficking dysfunctionality.

The combination of selective with permissive positional substrate specificity of BACE1 [31,32] (discussed in next section), is suggestive of substrate diversity and multiple specialized functions for BACE1 relative to generalized proteolytic function of the majority of other human aspartic proteases. Numerous BACE1 substrates, many of which are validated (reviewed in [73]), have been identified from deductive investigations and screening studies employing biochemical, cellular and molecular biology, as well as proteomic and genetic methods [74,75,76,77,78,79,80,81,82,83,84]. In particular, several substrates relate to processes and functions in the central nervous system (CNS; recently reviewed in [85]), including neuregulin 1 (Nrg1) type I and III-β1α, neuregulin 3, seizure protein 6, and sodium gated voltage channel β2 (Navβ2).

Peripheral nerves in Nrg1-deficient mice are hypomyelinated [86,87], a phenotype mimicked in mice with *BACE1* gene deletion [77], and additionally observed in regenerating peripheral neurons [78,88]. Moreover, developmental myelination effects of *BACE1* deletion are evident in the CNS, accompanied with significantly increased sensitivity to pain and reduced grip strength [78]. However, effects of *BACE1* deletion on CNS developmental myelination were found to be less evident in other studies but not investigated to the same extent [77,89]. Although types I and III Nrg1 are substrates of ADAM10 [90], it does not provide a functional redundancy, and is nonessential, substantiating BACE1 activity as a critical step in Nrg1-mediated myelination. BACE1 additionally cleaves substrates Jag1 and Delta1 to control proliferation and proper function of Schwann cells in axon myelination in the periphery [73,91], furthering the evidence for role of BACE1 in peripheral nerve myelination throughout lifetime.

Further implicating a specific role of BACE1 in the CNS, the processing of Navβ2 [79,92] results in reduced secretion of the Navβ2 to the plasma membrane and reduced sodium current densities that result in hyperexcitability to an extent consistent with seizures observed in BACE1 knockout animals [93]. Additionally, BACE1 was found to interact non-catalytically with the main α subunits of sodium and potassium channels and may serve as the β subunit [93,94,95]. Thus, interpretation of BACE1 gene depletion would impact both roles of BACE1 in cellular excitability, whereas inhibition of BACE1 would reduce processing of β subunits, with less expectation to impact BACE1 participation as a β subunit where its catalytic activity has been demonstrated to be dispensable [93,95].

Directly related to seizure phenotype observed in mice deficient in BACE1, Sez6 and Sez6L were identified [81] and validated [84] as BACE1 substrates, with Sez6 being implicated in susceptibility for febrile seizures [96]. Although seizures have not been observed in Sez6 KO mice, complicity with other deficient products in the CNS resulting from BACE1 protein depletion may enable emergence of the phenotype [73,97,98].

Mounting evidence supports a role of BACE1 in promoting neuroinflammation in AD. Proinflammatory cytokine IL-1 is secreted by microglia as well as astrocytes, endothelial cells, infiltrating leukocytes, neurons and oligodendrocytes [99] and induces secretion of additional proinflammatory cytokines from astrocytes and microglia. The IL-1 decoy receptor IL-1R2, that binds to IL-1 but does not possess capability for downstream activation, was demonstrated to be secreted by BACE1 activity [100]. Increased BACE1 expression and activity in AD would be expected to lead to increased IL-1R2 secretion (sIL-1R2); however, sIL-1R2 binds IL-1 with high affinity. Although enhanced inflammatory effects of IL-1 have been implicated in AD, and increased sIL-1R2 has been identified in early but not late AD [101,102], effects are not clear [103]. Nonetheless, the shedding of sIL-1R2 by BACE1 further implicates BACE1 in the AD process, although with uncertainties resulting from BACE1 inhibition at early or late stages of AD.

### 2.3. BACE1 Substrate Specificity: Implications for A673T

Amino acid sequences in the proximity of the cleavage site for a selection of validated BACE1 substrates [73] are listed in Table 2, organized by the allotment of amino acids on each side of the scissile (cleavage) bond at each position of the protein substrate (numbered from amino to carboxy terminus: P4, P3, P2, P1, P1’, P2’, P3’, P4’). Additionally, the sequence of the APP A673T protective mutation is included. All substrates feature generally hydrophobic amino acids at P1 and P3 sites, negatively charged at P1’, generally in accord with complete specificity determinations of BACE1 for peptide substrates [31,32], with specificity increasingly permissive for positions more distal to the scissile bond. 

In particular, the Thr present at P2’ in APP protective mutant A673T [15] is predicted to have subtle reduction (approximately 10%–20%) in catalytic efficiency relative to native APP [31,32], although isolated synthetic substrate was with approximately 5-fold lower *V*_max_ and unchanged *K*_M_ [104]. Nonetheless, secretion of Aβ from cells expressing APP A673T was reduced by approximately 40% [15] and presumably 20% in carriers [105]. Although the magnitude of Aβ reduction in plasma may not reflect that in the brain [106], a 28% reduction of plasma Aβ in carriers of the APP A673T gene compared to control subjects [107] is consistent with the biochemical studies, and supportive of a beneficial impact of reduced Aβ. Additionally, the protective effect of A673T may also be dependent on the efficiency of gamma secretase to process the A673T APP C-terminal fragment (C99) substrate that results from BACE1 cleavage [108]. Therefore, it might be considered that a combination of both BACE1 and gamma-secretase catalytic efficiencies may contribute to the decrease in Aβ production observed from in vitro and human subjects. Additional potential benefits resulting from the protective A673T APP mutation results from the gene product Aβ peptide having a Thr amino acid at position 2 in the sequence, and was found to have less propensity for aggregation [104]. Also of note, the threonine at position 2 of the A673T Aβ is adjacent to a native glutamate at position 3 (Table 2). An aminopeptidase activity may remove two amino terminal residues to create truncated Aβ species with a glutamate amino terminus [109], which are subsequently cyclized by the action of glutaminyl cyclase [110,111] to form highly insoluble and aggregation-prone pyroglutamate Aβ peptides (pyroGluAβ [112,113]; see next section). The activity the aminopeptidase activity that generates Aβ_3-x_ substrates for glutaminyl cyclase might be impacted by the Ala to Thr change in the P1 position of A673T-generated Aβ. Thus, in addition to the estimated 20–40% reduction in Aβ production [15,105] and slight reduction in aggregation properties [104], may be enhanced or offset by changes in the production rate of pyroGluAβ.

A mutation in APP at the same P2’ position of the β cleavage site is associated with FAD, A673V, and was predicted and observed to result in increased BACE1 activity [31,32,104], consistent with increased production of Aβ contributing to rapid accumulation of amyloid [104]. Notably, Thr is present at P2’ for substrates ST6Gal, Nrg1, and IL-1R2 (Table 2, [73]), and Val is present for several of the substrates in Table 2, although offsetting contribution of amino acids in other positions affecting catalytic efficiency must be taken in account. Interestingly, although the P2’ site was not found to be particularly restrictive [31,32], the P2’ site of the aligned cleavage sites of the substrates in Table 2 reveals to be the most restrictive, with only four amino acids found to be present at this position in this small set of substrate sequences.

## 3. The Prospect of Therapeutic Inhibition of BACE1 Activity to Block Aβ Production

### 3.1. Aβ

Aβ in various forms including soluble oligomers [114,115] disrupt synaptic transmission and cognition, induce neurotoxicity, and contribute to the overall disease process [7,16,115,116]. Increased level of amyloid is implicated as an early factor in AD [4], through increased production [116] or reduced clearance [117], with a resulting imbalance in the production and consumption “Aβ economy” [13]. Alternatively, Aβ may function beneficially in synaptic transmission [9,10], synaptic feedback inhibition [11,12], and even as an anti-microbial peptide [118,119]. Despite these and other possible functional roles for Aβ, increased amyloid and forms of Aβ resulting in downstream pathologic events might result from a dysregulated environment for an otherwise beneficial function [9,120].

A myriad of Aβ peptides (referred to as Aβ for simplicity) are generated from APP by the sequential action of β- (BACE1) and γ-secretases, and post-translational modifications [1], including notably pyrGluAβ, a major constituent of amyloid plaque with greatly higher propensity for aggregation [109,110,111,112]. Subsequent oligomerization and seeding events result in production of a further diverse set of peptides, with synaptotoxic, cognitoxic and neurotoxic effects.

Oligomeric forms of Aβ may directly interact with neuronal receptors [121,122,123,124,125,126], inhibiting their function or inducing downstream synaptoxicity. Inhibiting BACE1 to block Aβ production would be expected to deplete the supply of nascent Aβ feeding into the modification, oligomerization and accumulation pathways, thus diminishing oligomeric and all forms of Aβ contributing to synaptic dysfunction and other downstream effects [127,128,129].

### 3.2. Stragetic Approaches to BACE1 Inhibition

Discovery of a natural inhibitor of pepsin from *Actinomycetes* strains [130] led to the hypothesis of transition-state isostere inhibition of aspartic proteases [131], and ultimately to approved or candidate drugs targeting aspartic proteases of HIV-1, renin, *Plasmodium* and other protozoa, and fungi [20,132]. These successful medicinal chemistry efforts led to rapid development of potent prototypic BACE1 inhibitors suitable for in vitro [30,133] and in vivo proof of concept studies [134]. The initial promise of tractability and druggability was challenged by the requirement to incorporate properties that facilitate penetration of cells and the blood brain barrier while maintaining potency [20,21,135] and other properties for absorption, distribution, metabolism and excretion [136]. Selectivity over other aspartic protease family members, either highly homologous BACE2 [137,138,139] or others with essential housekeeping functions [17,140] was an additional hurdle. To overcome issues of selectivity, a biologics approach has been directed to BACE1, with modest success for either active or passive immunotherapies [141,142,143,144], including targeting the β cleavage site of APP [145,146].

The production of Aβ is blocked by BACE1 inhibition, resulting in reduced production of Aβ by depleting the supply of C99 substrate to gamma secretase. The BACE1 substrate APP is alternatively diverted to the non-amyloidogenic alpha secretase cleavage of APP and the resulting C83 substrate is cleared by gamma secretase producing the shortened P3 peptide (Aβ_17-40_, Aβ_17-42_ and others). P3 may not oligomerize [147] and is considered non-amyloidogenic. However, overproduction of P3 resulting from BACE1 inhibition and increased alpha secretase activity may not be without some unwanted effect; P3 is highly insoluble and was found to be a major component of diffuse plaque [148,149] and may impart other deleterious functions [149,150,151,152]. Alternatively, blocking cleavage of APP by BACE1 would preserve full-length APP which may serve functions at synapse including cell adhesion, neuronal migration and neurite outgrowth [153,154,155].

Extant Aβ is not reduced by BACE1 inhibition, and therefore reduction of Aβ is achieved by innate clearance mechanisms, including phagocytosis [156], transport proteins [156,157], and a growing list of candidate proteases that act upon soluble and insoluble forms of amyloid [158]. Impaired clearance of Aβ in late onset AD patients has been observed [117], presumably contributing to amyloid accumulation. Increases in BACE1 have been observed [159,160,161] that could be consistent with increased Aβ production at some stage in the disease, as BACE1 catalyzes the rate-limiting initial cleavage of APP [14]. BACE1 inhibitors have demonstrated reduction of Aβ in mild to moderate AD [14], suggesting that clearance, although possibly impaired at that stage of AD, is adequate to achieve Aβ reduction acutely, following from BACE1 inhibition [105]. However, lack of clinical benefit for BACE1 inhibitor verubecestat and for many Aβ-lowering drugs in mild to moderate AD [162,163] suggests that despite successful pharmacodynamic effect in amyloid reduction, a more relevant strategy may be reduction of Aβ production early in the disease process, or as a prophylactic, and is being explored for all BACE1 inhibitors currently in trials ([4,164,165]; see Table 4 in the section below).

### 3.3. Impact of BACE1 Inhibition on Other Biologic Functions

The discovery of multiple substrates of BACE1, in particular in the CNS, has continued to raise question of the acute and chronic effects of inhibiting BACE1 to block Aβ production while impeding the processing of other BACE1 substrates. To that end, nonclinical models including overexpression, partial, and complete deletion of *BACE1* have served to guide the clinical experience, summarized in Table 3 from several reviews [14,16,73,166] in context of the extent of depletion of BACE1 activity from either genetic or pharmacologic depletion.

The possible physiologic impairment of reduced native BACE1 biologic function may depend on the timing and target magnitude of Aβ reduction as discussed in the next section. Additionally, emergence of effects may depend upon challenging events (e.g., infection, injury, etc.). BACE1 inhibitors tested in the clinic ([14,29]; Table 4) generally do not exhibit effects (Table 3), although off-target effects related to either the chemical class or a particular BACE1 inhibitor drug, might add or compound effects and severity [167].

The primary goal during drug development is to assure safety of subjects and patients, with less emphasis placed on the pharmacologic benefit in early drug development [168]. Safety pharmacology and toxicity studies provide information for ranges of safe and tolerable dose administration, and knowledge of target organs and markers that provide sentinels for potential adverse events in the clinic [169]. These regulated studies model the route, frequency and duration of administration, with developmental stage or age appropriate to the target patient population [169]. The BACE1 deficiency studies (Table 3 and reviews referenced) span from embryonic development throughout adult life and therefore may not be suitable for comparison to studies that evaluate the toxicity or safety of a treatment regimen of a particular compound [169]. For example, BACE1 participation as a β subunit in sodium and potassium channels would be impacted by BACE1 depletion but may not be expected to be impacted by inhibition of BACE1 proteolytic activity ([93]; discussed in section above). Additionally, differences in the extent of pharmacologic inhibition may explain to some extent the discord between KO and pharmacologic inhibition studies in functional CNS outcomes [14,29]. However, the battery of work (Table 3) has demonstrated a complex biology for BACE1 and should continue to provide invaluable guidance to the understanding of BACE1 biology and to the development of BACE1 inhibitors.

### 3.4. Magnitude of Aβ Reduction to Prevent the Onset of AD

Several BACE1 inhibitors have advanced to Phase 3 clinical trials in asymptomatic, early or prodromal AD (Table 4; [14,73,105,163,166,184,185]). Despite their varying biochemical, specificity, and pharmacokinetic properties, maximal pharmacodynamic effects of lowering Aβ in the CSF by >90% have been demonstrated for reduction of various Aβ species (Aβ_1-x_ or Aβ_x-40_, Aβ_x-42_) in early Phase 1 trials for all compounds in Table 4. The pharmacodynamic activity of these compounds and other BACE1 inhibitors tested in Phase 1 trials attest to the clinical suitability of BACE1 as a suitable target for Aβ reduction.

The BACE1 programs referenced in Table 4 seek 50% or greater reduction in Aβ production to demonstrate a preventative therapeutic benefit to thwart progression in asymptomatic, prodromal, or early AD. Evidence has been observed for cognitive and pathologic improvement in reducing Aβ by BACE1 depletion in mice that overexpress Aβ [16]. However, the overproduction of Aβ in many of these models may be many-fold excessive, or even nonexistent in AD [117]. Therefore the magnitude of reduction in Aβ production to achieve cognitive efficacy in these models, as either percentage or molar amount, might be considered to be model dependent.

Variations have been seen in reported concentrations of Aβ in CSF, and validated analytical methodologies are evolving [191]. In a recent study, baseline CSF concentrations of Aβ_40_ and Aβ_42_ were found to vary significantly between individuals, despite being relatively stable [192], with concentrations approximately in the range of 600–1200 pM for Aβ_40_ or 40–80 pM for Aβ_42_. Within such a wide range of variation, the prospect of seeking 50–75% reduction may result in Aβ levels lower than concentrations demonstrated to provide a beneficial effect [9,10] including concentrations demonstrated to provide beneficial anti-microbial effects [118,119,193]. Additionally notwithstanding, seeking inhibition of BACE1 to achieve the >50% magnitude of Aβ reduction may increase the risk of possible undesired effects from inhibiting BACE1 processing of other substrates. The possibility might be considered that depletion of Aβ below beneficial or protective levels might not have been biologically challenged in toxicity and safety studies, or in gene deletion studies.

## 4. Discussion

Maximally limiting the amount of competing nascent Aβ by an extensive magnitude of BACE1 inhibition must necessarily accelerate amyloid clearance. However, in a model of therapy in early AD or prevention of AD, slowing of accumulation seems to be more relevant than rapid clearance, where amyloid is in early stages of formation and accumulation is incomplete [4,6]. Thus it might be conceivable that extensive reduction in Aβ production and BACE1 activity might be considered unnecessary or possibly unbeneficial. Additionally, increased risk of possible associated undesirable effects both acutely and chronically might be avoidable with a less extensive regimen. However, the alternative of achieving a modest Aβ reduction may be challenging due to the larger variation in lower ranges of pharmacologic effect (for example, [29]).

The target 50–75% reduction of Aβ in the ongoing generally preventative/early AD trials of BACE1 inhibitor drug candidates (Table 4) contrasts to an approximated 20–40% protective reduction resulting from the A673T mutation [15,104,105,107]. Although production and inhibition of Aβ in plasma may not reflect that in the brain [106,189], the 28% reduction in plasma Aβ observed for A673T carriers [107] is aligned with the anticipated approximate 20% reduction in biochemical activity for Aβ production [15,104,105].

The approximate 20–40% reduction of Aβ production by A673T APP may be considered to be protective immediately prior to the onset of amyloid accumulation [4,6,105] and likewise tolerable to native functions of Aβ and APP processing [9,10]. The failure of all clinical trials testing amyloid interventions in mild-to-moderate AD has been attributed to many factors including reliance on nonclinical models that are not predictive of efficacy [194]. It might be considered that the scientific evidence from the A673T human experience could be a useful perspective to contextualize conclusions which will emerge from the range of Aβ reductions currently sought in Phase 3 clinical trials (Table 4).

Alternatively, it might also be considered that the modest magnitude of Aβ reduction resulting from protective A673T may provide an inaccurate target. The resulting altered Aβ may have effect on other Aβ pathways independent or downstream of the modestly reduced BACE1 APP processing activity. In particular, the protective aspect of a 20–40% reduction in Aβ reduction might be overestimated owing to the reduction in aggregation/oligomerization of A673T Aβ species [104]. Additionally, production of pyrGluAβ species might be reduced if the amino acid change at position 2 in Aβ impedes the generation of Aβ_3-x_ substrates for glutaminyl cyclase [110,111], reducing the impact of this highly aggregation prone Aβ species [112,113]. Importantly, the mutation has not been observed in other populations (for example, [195]), although the prospect of other protective mutations in the amyloid pathway that would be instructive of amyloid reduction has not been explored.

The observation of BACE1 involvement in numerous normal physiologic functions has raised the question of the impact of BACE1 inhibition on these and other as yet unknown biologic processes mediated by BACE1 [16,73]. Additionally, possible beneficial functions of Aβ at the synapse [9,10,11,12] and in anti-microbial activity [118,119,193] may be affected by depletion of Aβ. Toxicology studies may accomplish the goal of establishing a safe dose range for a BACE1 inhibitor but may not be of duration or sensitivity to discern BACE1 biology, which is equally important in continued monitoring of patient safety. Continuing scientific investigations of BACE1 substrates and physiology [16,73] including the continued search for BACE1 substrates and the impact of chronic administration of BACE1 inhibitors on specific pathways at relevant stages of AD will be expected to aid continued safety vigilance of BACE1 inhibitor development.

## Figures and Tables

**Table 1:** Post-translational modifications observed for BACE1 ^1^.

Feature	Position in Amino Acid Sequence ^2^	Function	References
Propeptide	22–45	Folding, enhancement of activity	[34,35,36]
Disulfide bonds	216–*420*, 278–*443*, 330–380	Structural stability	[30]
Glycosylation	153, 172, 223, 354	Lysosomal targeting, degradation	[37,38,39]
Phosphorylation	*498*	Endosomal-lysosomal trafficking	[40]
Ubuiquitination	*501*	Trafficking, degradation	[41,42]
S-Palmitoylation	*474*, *478*, *483*, *485*	Lipid raft, amyloidogenesis	[43]
Lysine acetylation	126, 275, 279, 285, 299, 300, 307	Stability; transit from ER; enhancement of intracellular activity	[37,44]

^1^ From [45] with modification; ^2^ Location in the BACE1 amino acid sequence (Uniprot entry P56817), numbered 1–501 (homologous pepsin domain spans amino acids 62–416, lumenal extension 417–454, hydrophobic region 455–480, and cytoplasmic domain 481–501); residues in italics are *C*-terminal to the homologous pepsin domain; conserved cysteine residues are underlined.

**Table 2:** BACE1 substrate amino acid sequences in the proximity of the BACE1 cleavage site ^1^.

				↓				
Substrate ^2^	P4	P3	P2	P1	P1’	P2’	P3’	P4’
APP, β site	E	V	K	M	D	A	E	F
APP A673T, β site	E	V	K	M	D	T	E	F
APP, β’ site	D	S	G	Y	E	V	H	H
Nrg1 type I & III-β1α	G	I	E	F	M	E	A	E
Nrg3	G	I	E	F	M	E	S	E
IL-1R2	T	L	S	F	Q	T	L	R
Navβ2, major site	L	Q	V	L	M	E	E	P
Navβ2, minor site	K	I	H	L	Q	V	L	M
Nrg1 type III-β1α	E	T	N	L	Q	T	A	P
Sez6	G	R	S	L	D	V	A	K
Delta-1	V	V	D	L	T	E	K	L
PSGL-1	A	S	N	L	S	V	N	Y
Jag2	S	L	L	L	A	V	T	E
Sez6L	A	L	E	A	E	A	A	A
Jag1	S	L	I	A	A	V	A	E
ST6Gal	E	K	A	Q	L	T	L	A
CHL-l	S	I	F	Q	D	V	I	E

^1^ From reference [73]; ^2^ Sequences (single letter amino acid code) are organized by hydrophobicity, charge, and size in P1 and P1’ positions; the ↓ symbol denotes the scissile (cleavage) bond; threonine present in P2’ are boxed.

**Table 3:** Morphologic, physiologic and behavioral impact of BACE1 depletion ^1^.

Function/Dysfunction	Degree of BACE1 Inhibition ^2^		Reference
	Pharmacologic, Subchronic	Gene Deletion	
Neurogenesis, Astrogenesis		100%	[170]
Growth cone collapse		100% ^3^	[171]
Axonal growth	100%	50–100% ^4^	[172,173,174]
Spine density	60% ^5^	100%	[175,176]
Muscle Spindle	68% ^6^	100%	[97]
Myelination		100% ^7^	[77,78,88]
Synaptic dysfunction	50% ^5^	100% ^7^	[176,177,178,179]
Retinopathy		100%	[180]
Muscle coordination		100%	[171,178,181]
Memory impairment	60% ^5^	100% ^7^	[176,177,178]
Lethality, growth impairment		50–100%	[98,181,182]
Seizures		100%	[97,98,178]
Social/emotional		100% ^6^	[177,182,183]
Psychosis		100%	[175,178]

^1^ Adapted from [73,166]; ^2^ 50% and 100% refer to heterozygous and homozygous *BACE1* gene deletion, unless otherwise noted for pharmacologic inhibition; ^3^ BACE1 null and ex vivo inhibition of BACE1 100%; authors suggest window of 100-fold for 40% Aβ reduction; ^4^ Trend in gene dosage; ^5^ Pharmacologic inhibition of 50% and 60% at 30 and 100 mg/kg doses of SCH1682496, respectively [176]; ^6^ Effects not evident in heterozygotes.

**Table 4:** Target Aβ reduction for BACE1 inhibitors currently in Phase 3 clinical trials for AD.

Compound	Sponsor(s)	Patient Population and Identifier ^1^	Dose, Target Aβ Reduction ^2^	Reference
Verubecestat (MK-8931)	Merck	Prodromal AD (NCT01953601)	12 mg: 50% 40 mg: 75%	[29]
Lanabecestat (LY3314814, AZD3293)	AstraZeneca, Eli Lilly	Early AD (NCT02245737)	20 mg: 60% ^3^ 50 mg: 75% ^3^	[186,187]
Elenbecestat (E2609)	Biogen Idec, Eisai	Early AD (NCT03036280, NCT02956486)	50 mg: 60% ^4^	[188]
JNJ-54861911	Shionogi, Janssen	Asymptomatic, at risk for AD (NCT02569398)	5 mg: 50% 25 mg: 75–85%	[189]
CNP520	Novartis, Amgen	At risk for AD (NCT03131453)	15 mg: N.D. ^5^ 50 mg: N.D. ^5^	[190]

^1^ ClinicalTrials.gov; ^2^ Average or AUC reduction in Aβ, to nearest 5%; ^3^ Maximum reduction, approximates AUC owing to extended pharmacodynamic effect of AZD3293; ^4^ Average or maximum not reported; ^5^ N.D., clinical pharmacodynamic activity not disclosed.
